# Assessment of Drug-Induced Toxicity Biomarkers in the Brain Microphysiological System (MPS) Using Targeted and Untargeted Molecular Profiling

**DOI:** 10.3389/fdata.2019.00023

**Published:** 2019-06-26

**Authors:** Sara G. Mina, Begum Alaybeyoglu, William L. Murphy, James A. Thomson, Cynthia L. Stokes, Murat Cirit

**Affiliations:** ^1^Department of Biological Engineering, Massachusetts Institute of Technology, Cambridge, MA, United States; ^2^Department of Biomedical Engineering, University of Wisconsin-Madison, Madison, WI, United States; ^3^Regenerative Biology, The Morgridge Institute for Research, Madison, WI, United States; ^4^Department of Cell and Regenerative Biology, University of Wisconsin, Madison, WI, United States; ^5^Stokes Consulting, Redwood City, CA, United States

**Keywords:** microphysiological systems, neurotoxicity, biomarkers, metabolomics, predictive models

## Abstract

Early assessment of adverse drug effects in humans is critical to avoid long-lasting harm. However, current approaches for early detection of adverse effects still lack predictive and organ-specific biomarkers to evaluate undesired responses in humans. Microphysiological systems (MPSs) are *in vitro* representations of human tissues and provide organ-specific translational insights for physiological processes. In this study, a brain MPS was utilized to assess molecular signatures of neurotoxic and non-neurotoxic compounds using targeted and untargeted molecular approaches. The brain MPS comprising of human embryonic stem (ES) cell-derived neural progenitor cells seeded on three-dimensional (3D), chemically defined, polyethylene glycol hydrogels was treated with the neurotoxic drug, bortezomib and the non-neurotoxic drug, tamoxifen over 14-days. Possible toxic effects were monitored with human N-acetylaspartic acid (NAA) kinetics, which correlates the neuronal function/health and DJ-1/PARK7, an oxidative stress biomarker. Changes in NAA levels were observed as early as 2-days post-bortezomib treatment, while onset detection of oxidative stress (DJ-1) was delayed until 4-days post-treatment. Separately, the untargeted extracellular metabolomics approach revealed distinct fingerprints 2-days post-bortezomib treatment as perturbations in cysteine and glycerophospholipid metabolic pathways. These results suggest accumulation of reactive oxygen species associated with oxidative stress, and disruption of membrane structure and integrity. The NAA response was strongly correlated with changes in a subset of the detected metabolites at the same time point 2-days post-treatment. Moreover, these metabolite changes correlated strongly with DJ-1 levels measured at the later time point (4-days post-treatment). This suggests that early cellular metabolic dysfunction leads to later DJ-1 leakage and cell death, and that early measurement of this subset of metabolites could predict the later occurrence of cell death. While the approach demonstrated here provides an individual case study for proof of concept, we suggest that this approach can be extended for preclinical toxicity screening and biomarker discovery studies.

## Introduction

Given that central nervous system (CNS) toxicity is a leading cause of toxicity-related clinical trial failures (Cook et al., 2014; Walker et al., 2018), the predictive capabilities of current pre-clinical toxicity testing methods remain inadequate. The two-dimensional (2D) mono-cultures of typical *in vitro* CNS models fail to recapitulate the physiological complexity of CNS tissues, limiting their ability to predict adverse responses at the tissue or organ level (*in vivo*) from the effects observed at the molecular or cellular level (*in vitro*) (Langhans, 2018). Further, while high-throughput screening (HTS) utilizing such 2D models enables rapid testing of large numbers of compounds, the endpoints used, such as live/dead staining and lactate dehydrogenase (LDH) release, are necessarily simple and limited in translational relevance. Animal models remain a critical screen, yet species differences limit their predictive capability. New methodologies are sorely needed to better predict human neurotoxic liabilities of new molecular entities (NMEs) rapidly and robustly prior to clinical trials.

The emerging field of microphysiological systems (MPSs) holds promise for this mission (Marx et al., 2016; Low and Tagle, 2017). MPSs encompass a range of cellular- and tissue-level models in three-dimensional (3D) culture platforms meant to recapitulate more physiologically-relevant functions of human organs and tissues compared to traditional 2D culture systems. MPSs are more cost-effective than animal models and can be used for numerous pharmaceutical development applications including drug absorption, distribution, metabolism, excretion, and toxicity (ADMET), evaluating efficacy and investigating pharmacodynamic mechanisms. For example, neural toxicity has been investigated in a brain MPS comprising a 3D construct of mixed neuronal and glial cells derived from human embryonic stem (ES) cells (Schwartz et al., 2015), and hepatic drug metabolism has been studied in a 3D human liver MPS (Tsamandouras et al., 2016). Likewise, drug absorption and metabolism processes have been studied in an integrated gut-liver platform that enables observation of organ-organ crosstalk (Chen et al., 2017; Tsamandouras et al., 2017). Higher degree multi-MPS systems have also been applied to assess systemic drug effects on human physiology (Maschmeyer et al., 2015; Oleaga et al., 2016; Zhang et al., 2017; Edington et al., 2018).

MPS technologies enable longer-term tissue culture to study kinetics of drug-physiology interactions using various continuous and endpoint metrics. Not only might they be used to screen out toxic molecules at the preclinical stage, but they could potentially provide insights for development and validation of clinical biomarkers. For example, omics-profiling (proteomics, metabolomics) combined with measurements of neuronal electrical activity in a mixed neuronal/glial cell culture platform identified several potential biomarkers of drug-induced neurotoxicity (Schultz et al., 2015). Early response biomarkers in humans that presage later overt toxicity would be particularly valuable. As of 2011, fewer than 100 biomarkers were validated for clinical use, highlighting the difficulties connected with translating scientific findings to clinical decision making (Poste, 2011).

Fluid-based molecular biomarkers such as those found in serum, plasma, urine, and cerebrospinal fluid (CSF) have the advantages of relative ease of sampling using minimally invasive methods, as well as the possibility of frequent or even continuous monitoring. Two biomarkers of neural function accessible in blood and urine include *N*-acetylaspartic acid or *N*-acetylaspartate (NAA) and DJ-1/PARK7 protein. NAA, the acetylated form of the amino acid aspartate, is one of the most abundant brain metabolites (Moffett et al., 2012), and has been shown to be an indicator of neural cell function, while human DJ-1, an oxidative stress indicator, is utilized for evaluating drug-induced neurotoxicity (Kahle et al., 2009).

Numerous studies have demonstrated that NAA levels are altered in the brain in a variety of human CNS disorders. Its concentration declines in nearly all, including Alzheimer's disease (Bittner et al., 2013; Murray et al., 2014), MS (Tortorella et al., 2011), and schizophrenia (Harris et al., 2006), although it is elevated in Canavan disease (Wittsack et al., 1996; Moffett et al., 2012) due to the lack of a metabolizing enzyme. NAA levels were increased in the serum of ALS patients, thought to be related to greater excretion of NAA into the circulation following release from damaged neurons (Simone et al., 2011).

DJ-1 is a ubiquitous redox-responsive protein, mainly localized in the cytosol and also found in mitochondria and the nucleus. Studies have shown that during oxidative stress, DJ-1 may modulate the expression of genes such as glutamate-cysteine ligase, which results in glutathione (GSH) metabolite formation (Kim et al., 2012). Biochemically, DJ-1 is easily oxidized in response to several oxidative stimuli, and the oxidized, acidic isoforms of DJ-1 have been found to be accumulated in the brains of patients with sporadic Parkinson disease and Alzheimer's disease (Choi et al., 2006).

In this study, drug-induced neurotoxicity using targeted and untargeted biomarkers was evaluated in the brain MPS. The effects of the chemotherapeutic compound bortezomib, a proteasome inhibitor and known neurotoxic drug (Badros et al., 2007; Argyriou et al., 2014; Canta et al., 2015), were assessed and compared to effects of the selective estrogen receptor modulator (SERM) tamoxifen, which has no clinically reported neural adverse effects (Ernst et al., 2002; Stouten-Kemperman et al., 2015; Hong et al., 2017). Neurotoxicity was first assessed with the targeted biomarkers NAA and DJ-1 protein. Then, untargeted metabolomics was used to identify additional early response biomarkers after the first drug dose and these were correlated with NAA and DJ-1 at different time points to identify whether they are predictive of known markers of neuronal function and toxicity measured at later times.

## Materials and Methods

### Neural Progenitor Cell Culture

Neural progenitor cells (NPCs) derived from the human H1 ES line developed in the laboratory of Professor James Thomson, Morgridge Institute, Madison, WI were provided for this study. The NPC derivation methods were previously described (Schwartz et al., 2015). NPCs were cultured at 37°C and 5% CO_2_ in neural expansion medium comprising DMEM/F-12 medium (500 mL) supplemented with rhFGF2 (5 ng/ml), 1X N2 (Life Technologies, Carlsbad, CA, USA), 1X B27 (Life Technologies), L-ascorbic acid-2-phosphate magnesium (64 mg/L, Sigma-Aldrich, St. Louis, MO, USA), Sodium/selenium (14 μg/L, Sigma), NaHCO3 (543 mg/L, Sigma), penicillin-streptomycin (10% v/v 10,000 units/ml, ThermoFisher, Waltham, MA, USA). Cryopreserved NPCs were passaged on T-75 flasks coated with Matrigel (growth factor reduced, Corning 356230, 0.1 mg/ml for 1 h) in neural expansion medium. NPCs were maintained in a humidified incubator at 37°C and 5% CO_2_ below 90% confluence on Matrigel-coated polystyrene flask before passage. Cells were passaged every 4–5 days using Corning Accutase Cell Detachment Solution, Liquid (ITC AT104, Mediatech, Manassas, VA, USA) and neural expansion medium to neutralize Accutase. Cells were then pelleted by centrifugation at 240 relative centrifugal force for 3 min and a cell count was performed using the Luna Countess™ II FL Automated Cell Counter (ThermoFisher Scientific, Waltham, MA, USA). NPCs were cryopreserved at 1.2 × 10^7^ cells per vial or harvested on MPS.

### Polyethylene Glycol (PEG) Hydrogel Formulation

Polyethylene glycol (PEG) hydrogels were formed using “thiol-ene” photopolymerization chemistry from previously published protocols (Fairbanks et al., 2014; Hansen et al., 2014). The PEG hydrogel solution was purchased from Stem Pharm, Inc. (Madison, WI, USA). Hydrogel formulation comprised 40 mg/mL 8-arm PEG-norbornene, 4.8 MMP-peptide crosslinker (9.6 M cysteine, 60% molar ration relative to norbornene arms), 2 mM CRGDS C-amidated peptide, and Irgacure 2959 photoinitiator in PBS. Gels were polymerized in 0.33 cm^2^ Transwell® inserts (Corning 3470, Corning Life Sciences, Teterboro, NJ, USA) with 40 μL total volume and 4.8 J/cm^2^ of 365 nm UV light (10 min, 8 mW/cm^2^).

### Drug Preparation

Lyophilized drugs were solubilized in dimethyl sulfoxide (DMSO) and drug stock solutions were prepared in neural expansion medium. Bortezomib (Sigma-Aldrich) was prepared in the dark by dissolving powder in 100% DMSO and sterile filtered to obtain a stock solution of 10 mM. 0.1% drug stock was serially diluted in media for final dosing concentrations ranging from 0.001 to 10 μM, increasing by factors of ten. Tamoxifen (LC Laboratories, Woburn, MA, USA) was prepared by dissolving powder in 100% DMSO and sterile filtered to obtain a stock solution of 10 mM. Drug stock was serially diluted in media for final dosing concentrations ranging from 0.01 to 10 μM, increasing by factors of ten.

### Formation of 3D Neural Constructs and Neurotoxicity Experiments

#### Seeding H1 ES Cell-Derived Neural Progenitor Cells (NPCs) on PEG Hydrogels

The NPCs were harvested for MPS seeding between passages 5 and 8. NPCs were seeded at density of 50,000 cells/0.33 cm^2^ Transwell® and cultured on the PEG gel for 14-days prior to drug exposure. NPC media was changed every 2-days using 200 μL in the apical compartment, 1 mL in the basal compartment.

#### Drug-Induced Neurotoxicity Experiments

After 14-days of culture to establish the brain MPS, the 3D neural constructs were exposed to chemotherapeutic compounds for another 14-days, with media exchanged every 2-days. The time reported for all results are relative to establishment of culture on day 0. The chemotherapeutic neurotoxic compound, proteasome inhibitor, bortezomib (low-dose: 0.001 μM, mid-dose: 0.01 μM, and high-dose: 0.1 μM), or non-neurotoxic compound, selective estrogen receptor modulator (SERM), tamoxifen (low-dose: 0.01 μM, mid-dose: 0.1 μM, and high-dose: 1 μM) were compared to untreated controls for 14-days. For context, the bortezomib clinical maximum concentration (C_max_) is ~0.6 μM (Moreau et al., 2012) and tamoxifen C_max_ is ~0.2 μM (Kisanga et al., 2004). The prepared drug concentrations in the neural expansion media were added to both the apical and basal compartments to avoid concentration gradients.

### Quantification of NAA and DJ-1/PARK7

N-acetylaspartate (NAA) concentration in media samples was measured and analyzed every 2-days as described previously (Edington et al., 2018).

Human DJ-1/PARK7 concentration was measured in media samples every 2-days using an electrochemiluminescence multi-array immunoassay run according to the manufacturer's protocol (Human DJ-1/PARK7 Kit, Meso Scale Diagnostics, LLC, Rockville, MD, USA) as follows. A 7-point calibration curve with 4-fold serial dilution steps and a zero calibrator blank were prepared in diluent buffer (Diluent 35). Media samples were thawed and diluted in media blanks (1:2 v/v samples/media). Plates were blocked, washed, and incubated with diluted samples then read on a MESO QuickPlex SQ 120 instrument. Data were analyzed with Meso Scale Diagnostics' Discovery Workbench software.

### Immunocytochemistry

Both 2D laminin-coated Transwell inserts and 3D neural constructs on Transwell inserts were fixed *in situ* with 4% paraformaldehyde (Sigma-Aldrich, St. Louis, MO, USA) and incubated for 15 min at room temperature. The samples were stained for neuron-specific β-III tubulin antibody (1:500 v/v, monoclonal mouse IgG; R&D Systems, Minneapolis, MN, USA) and glial fibrillary acidic protein (GFAP; 1:500 v/v, goat polyclonal to GFAP, Abcam, Cambridge, MA) in incubation buffer (0.05% Triton X-100 and 1% bovine serum albumin in phosphate buffered saline), followed by overnight incubation at 4°C. Then, the cells were washed 2 times for 60 min with rinse buffer (0.05% Triton X-100 in phosphate buffer saline), followed by an overnight rinse at 4°C in rinse buffer. After rinse, 1:200 dilutions of secondary antibodies (Alexa Fluor® 488, Alexa Fluor® 568, and Hoechst 1:1000 v/v, Invitrogen, Carlsbad, California, USA) were added, followed by incubation overnight at 4°C or at least 4 h in the dark at room temperature. After that, the cells were rinsed and incubated overnight at 4°C in rinse buffer. Fluorescent images were obtained using a Keyence confocal imaging system with a 20x objective.

### Measurement of Untargeted Metabolomics for Drug-Induced Neurotoxicity

Media samples at day 16 (2-days post-treatment) for cells treated with 0.001–0.1 μM bortezomib, and 0.01–1 μM tamoxifen as well as untreated control were shipped for analysis to Human Metabolome Technologies America, Inc. (HMT; Boston, MA, USA). For sample preparation, 40 μL of samples were mixed with 10 μL of Milli-Q water containing internal standards (1,000 μM). The mixture was then filtered through a 5 kDa cut-off filter (ULTRAFREE-MC-PLHCC, Human Metabolome Technologies, Yamagata, Japan) to remove macromolecules. Metabolome analysis was performed in samples of culture medium using Capillary Electrophoresis Time-of-Flight Mass Spectrometry (CE-TOFMS) in two modes for cationic and anionic metabolites. On the basis of HMT's standard library, 65 metabolites (47 metabolites in Cation mode and 18 metabolites in Anion mode) were detected (dataset provided in Supplementary Materials).

### Analysis of Biomarker and Untargeted Metabolomics Data

The measured DJ-1 and NAA levels are reported as mean ± SD. The data were analyzed using a two-way ANOVA with Bonferroni's *post-hoc* testing for *n* = 3, *p* < 0.05 using GraphPad Prism version 7 (San Diego, CA, USA). For analysis of untargeted metabolomics, MetaboAnalyst R package (Chong et al., 2018) and Ingenuity Pathways Analysis (IPA, QIAGEN Bioinformatics, Redwood City, CA, USA) (Krämer et al., 2014) were used for multivariate and cluster analysis, and functional and pathway analysis, respectively. Reported metabolomics relative peak area data were normalized via auto scaling prior to statistical analysis. Altered metabolites were analyzed using multiple *t*-tests (individual *t*-tests for each metabolite) with Holm-Sidak correction and plotted using GraphPad Prism. Pathway maps were constructed in PathVisio desktop (Kutmon et al., 2015) for visualization of connectivity of the detected metabolites.

### Biomarker-Metabolite Correlation Analysis and Regression Models

Prior to correlation analysis, untargeted metabolomics data was refined by removing the metabolites that were not detected (N.D.) in the majority of the samples. For those metabolites that were either not detectable or became detectable upon drug exposure, relative abundance values were assigned as 2-fold lower than the lower limit of detection (LLOD/2). Lower limit of detection was defined as the lowest relative abundance value measured in any metabolite across the samples. Preprocessed untargeted metabolomics data was used as the input for all correlation analyses and regression models. Biomarker-metabolite correlation analyses were carried out and plotted using GraphPad Prism. Correlations with a coefficient larger than 0.7 (|*r*| > 0.7) were considered strong correlations and simple linear regression (SLR) models were employed for those correlated metabolite-biomarker couples. The regularized regression method Lasso (Tibshirani, 1996) was employed using the *caret* (Kuhn, 2008) and *glmnet* R packages (Friedman et al., 2010) to select the important metabolites (predictors from the intercorrelated metabolomics data; multicollinearity) to predict DJ-1 (dependent variable). The hyperparameter lambda (λ) was optimized at the minimum root mean square error (RMSE_min_) of the repeated 5-fold cross-validations (100 repetitions) over the whole dataset (R script in Supplementary Materials). The final model for prediction was constructed using the regression coefficients calculated at the optimized lambda. The predicted DJ-1 levels were then compared with the measured DJ-1 levels to calculate to goodness of fit (*R*^2^).

## Results

### Brain Microphysiological System (MPS) Supports Mixed Neural Cell Culture for 28-Days

**Figure 1:**
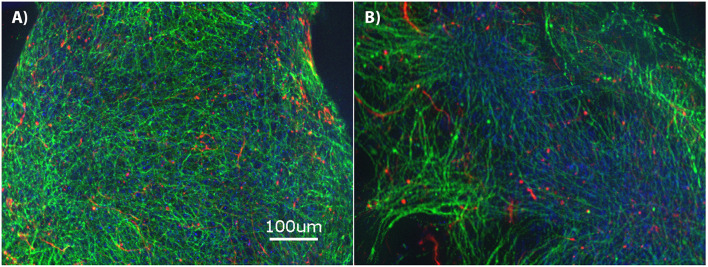
Brain microphysiological system (MPS): Neural progenitor cells (NPCs) cultured on three-dimensional (3D) chemically-defined poly(ethylene glycol) hydrogel (multilayer) at **(A)** 14-days and **(B)** 28-days. Immunocytochemistry images for β-III tubulin (neurons; green), Glial Fibrillary Acidic Protein (GFAP: glial cells; red), and Hoechst (nuclei; blue). Scale bar = 100 μm.

The brain MPS comprises a 3D PEG hydrogel with NPCs that have differentiated into βIII-tubulin^+^ (neurons) and GFAP^+^ (glial) cells and self-assembled to form 3D neural constructs. The mixed neuron-glial cell culture can be maintained in this environment for at least 28-days, as demonstrated by the immunofluorescence images in Figure 1A at day 14 and Figure 1B at day 28. Structurally, the images illustrate cells extending around the circumference of the 3D hydrogels.

### Assessment of Brain MPS Response to Bortezomib Using Targeted Biomarkers

**Figure 2:**
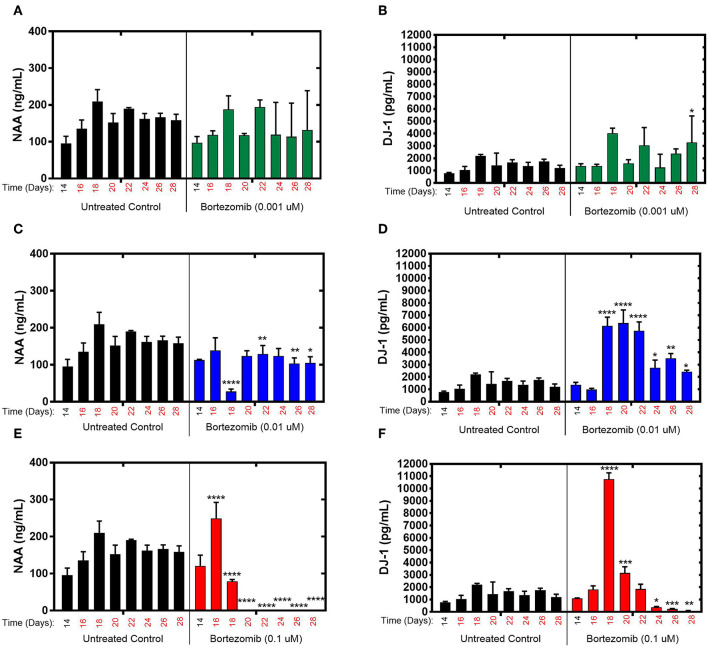
Bortezomib treatment for 14-days in the brain MPS. **(A,C,E)** Extracellular N-acetyl-aspartate (NAA) concentration in the media for the noted dose **(B,D,F)** Extracellular DJ-1/PARK7 concentration in the media for the noted dose. Each bar represents mean ± SD for triplicate MPS wells (*n* = 3) at each treatment dose and time point. For each bortezomib dose, significant differences between treated (colored bars) and untreated control (black bars) at the same time point are denoted according to the calculated *p*-values (two-way ANOVA multiple comparisons with a Bonferroni's *post-hoc* test; ^*^p < 0.05, ^**^p < 0.01, ^***^p < 0.001, and ^****^p < 0.0001). Day 14 represents the time at which drug dosing was initiated.

During exposure to bortezomib from days 14–28, NAA and DJ-1 levels were measured every 2-days to assess the drug-induced neurotoxicity in the brain MPS. Low-dose (0.001 μM) bortezomib induced no significant difference in NAA and DJ-1 levels compared to the untreated controls, as shown in Figures 2A,B. For 0.01 μM dose (mid-dose) of bortezomib on day 18 (4-day treatment), NAA levels were significantly reduced compared to control, and remained so at day 22 and beyond. In contrast, DJ-1 levels significantly increased and remained elevated for the duration of the drug exposure (Figures 2C,D). These results suggest that 0.01 μM bortezomib exposure leads to neurotoxicity in the brain MPS.

For high-dose (0.1 μM) bortezomib, on day 16 (2-day treatment) NAA levels were upregulated compared to the untreated control (Figure 2E), falling below control at day 18, and from day 20 (6-day treatment) through last day of treatment, falling below the limit of quantification, suggesting acute neuronal damage in that time period. In comparison, DJ-1 levels were at first unaltered, but then increased sharply by day 18, coinciding with the decline in NAA, and subsequently DJ-1 significantly decreased for the duration of treatment, compared to untreated controls, as shown in Figure 2F. Measurements and *t*-test results of bortezomib-treated brain MPSs are tabulated in Supplementary Table 1.These results demonstrated that changes in NAA and DJ-1, clinically-relevant biomarkers, in the brain MPS upon neurotoxic drug treatment were dose- and time-dependent.

### Assessment of Brain MPS Response to Tamoxifen Using Targeted Biomarkers

**Figure 3:**
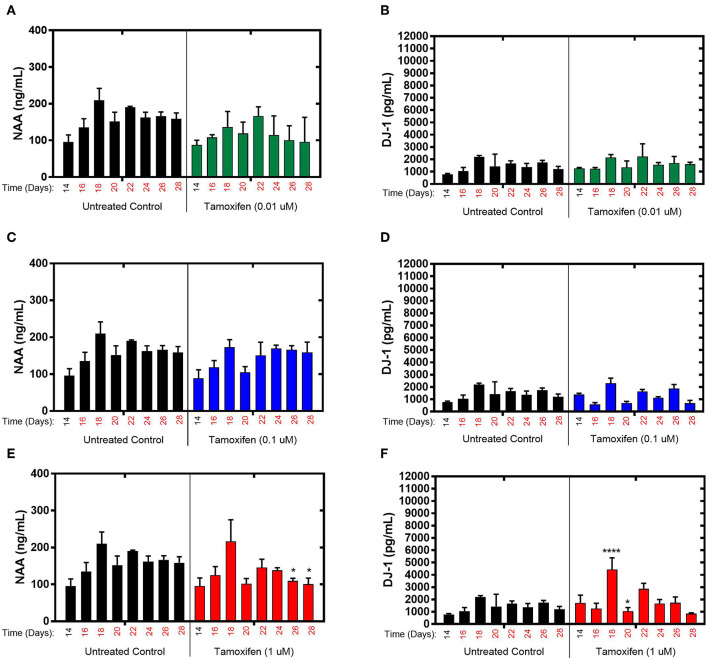
Tamoxifen treatment for 14-days of the brain MPS. **(A,C,E)** Extracellular N-acetyl-aspartate (NAA) concentration in the media for the noted dose **(B,D,F)** Extracellular DJ-1/PARK7 concentration in the media for the noted dose. Each bar represents mean ± SD for triplicate MPS wells (*n* = 3) at each treatment dose and time point. For each tamoxifen dose, significant differences between treated (colored bars) and untreated control (black bars) at the same time point are denoted according to the calculated *p*-values (two-way ANOVA multiple comparisons with a Bonferroni's *post-hoc* test; ^*^p < 0.05 and ^****^p < 0.0001). Day 14 represents the time at which drug dosing was initiated.

The effects of tamoxifen, a SERM with no clinically observed neurotoxicity in humans, on NAA and DJ-1 levels were tested in the brain MPS between days 14–28 in the brain MPS. For 0.1 μM (low-dose) and 0.01 μM (mid-dose) treatments, NAA and DJ-1 levels were not significantly different compared to untreated controls at any time point during treatment (Figures 3A–D). For 1 μM (high-dose) tamoxifen exposure, NAA levels were slightly reduced at days 26 and 28 (after 12- and 14-day drug exposure, respectively), as shown in Figure 3E. In comparison, DJ-1 level was elevated after 4-days of drug exposure (day 18), fell below untreated control at day 20, and then returned to levels similar to untreated control for the duration of the experiment (Figure 3F). In summary, the effects of tamoxifen on NAA and DJ-1 levels in the brain MPS are limited in comparison to those observed for bortezomib. Measurements and *t*-test results of DJ-1 and NAA levels of tamoxifen-treated brain MPSs are tabulated in Supplementary Table 1.

### Bortezomib and Tamoxifen Treatments Induce Distinct Metabolic Biomarker Profiles in the Brain MPS

**Figure 4:**
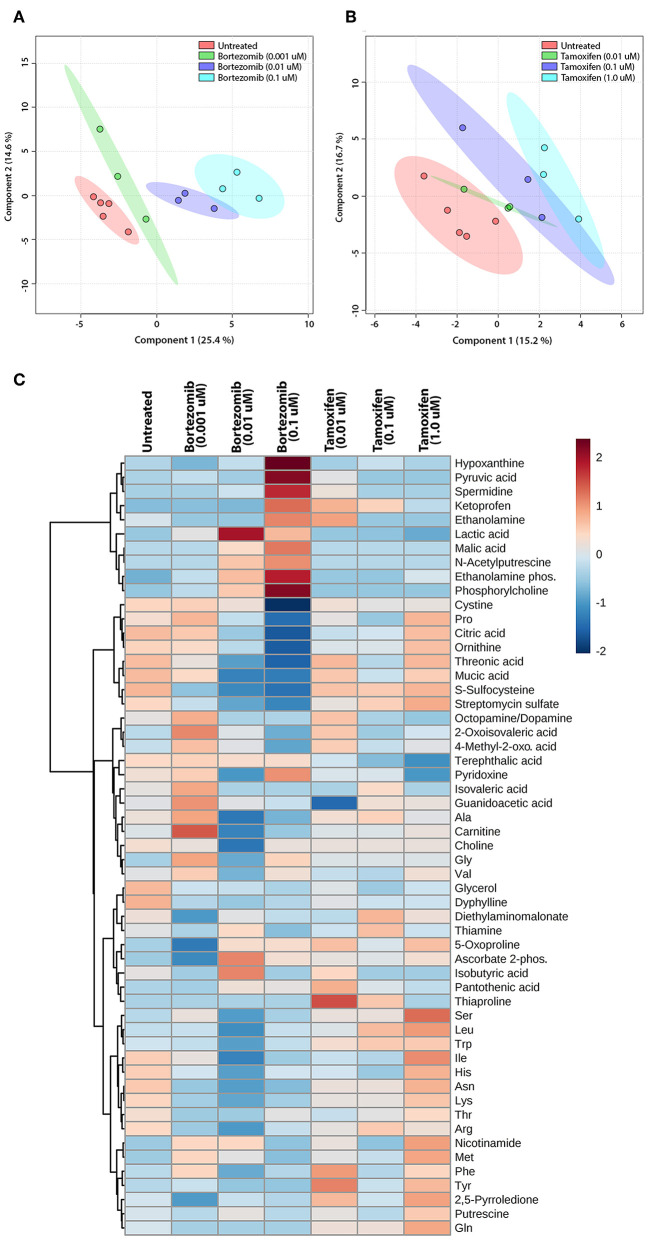
Multivariate and cluster analysis for untargeted metabolomics. PLS-DA scores plots for the **(A)** bortezomib or **(B)** tamoxifen treated groups (green, blue, and cyan circles represent low-dose, mid-dose, and high-dose, respectively, with their untreated controls in red circles, all within their 95% confidence intervals) showing the differences in metabolic fingerprints. **(C)** Heatmap of relative levels of the detected metabolites showing the untreated group (*n* = 5) compared to bortezomib or tamoxifen treated groups (*n* = 3 for each dose).

The untargeted metabolomics of extracellular media samples were investigated 2-days post-treatment for bortezomib and tamoxifen, and were compared to untreated controls. A total of 65 metabolites were detected in the media samples, and relative abundance values were used to indicate drug-induced alterations in the metabolic fingerprints. For the multivariate analysis, partial least squares discriminant analysis (PLS-DA) was carried out to visualize differences among the groups with respect to untreated controls. The PLS-DA scores plots revealed the separation between the different dose treatments of bortezomib (Figure 4A) or tamoxifen (Figure 4B) compared to untreated controls. Bortezomib treatment induced a more pronounced dose-dependent deviation from the untreated controls than did tamoxifen treatment, as indicated by data more closely packed and confidence regions overlapping in comparison with the untreated controls for tamoxifen. 10-fold cross validation for both PLS-DA models showed that *Q*^2^ was 0.79 for the first 2 components of the bortezomib model with *R*^2^ of 0.97 (Supplementary Table 2). For the tamoxifen model, calculated *Q*^2^ values were negative for up to 8 components, suggesting that the model is either not predictive or is overfitted (Supplementary Table 2).

The relative metabolite levels in the untreated controls and the drug-treated neural constructs are compared in the heatmap (Figure 4C) to detect the metabolites altered by drug treatment. The group averages (*n* = 3 for each drug and dosing) of the metabolic profiles suggested major differences in some metabolite levels in the mid-dose and high-dose bortezomib-treated groups compared to the untreated controls, while the low-dose group appeared comparable to the untreated group. In the mid-dose and high-dose tamoxifen-treated groups, alterations in some amino acid levels were detected, but the changes were not statistically significant (not shown). The low-dose tamoxifen treated group was similar to untreated controls.

To assess the significance of altered metabolites upon bortezomib or tamoxifen treatments, statistical analysis (multiple *t*-tests) was carried out. None of the metabolite alterations in the low-dose bortezomib treated group compared to untreated control were statistically significant, while perturbations in the levels of 3 metabolites in the mid-dose treated group, and of 5 metabolites in the high-dose treated group were statistically significant (*p*_adj_ < 0.05) compared to the untreated controls. For the tamoxifen-treated groups, none of the metabolite alterations were statistically significant.

**Figure 5:**
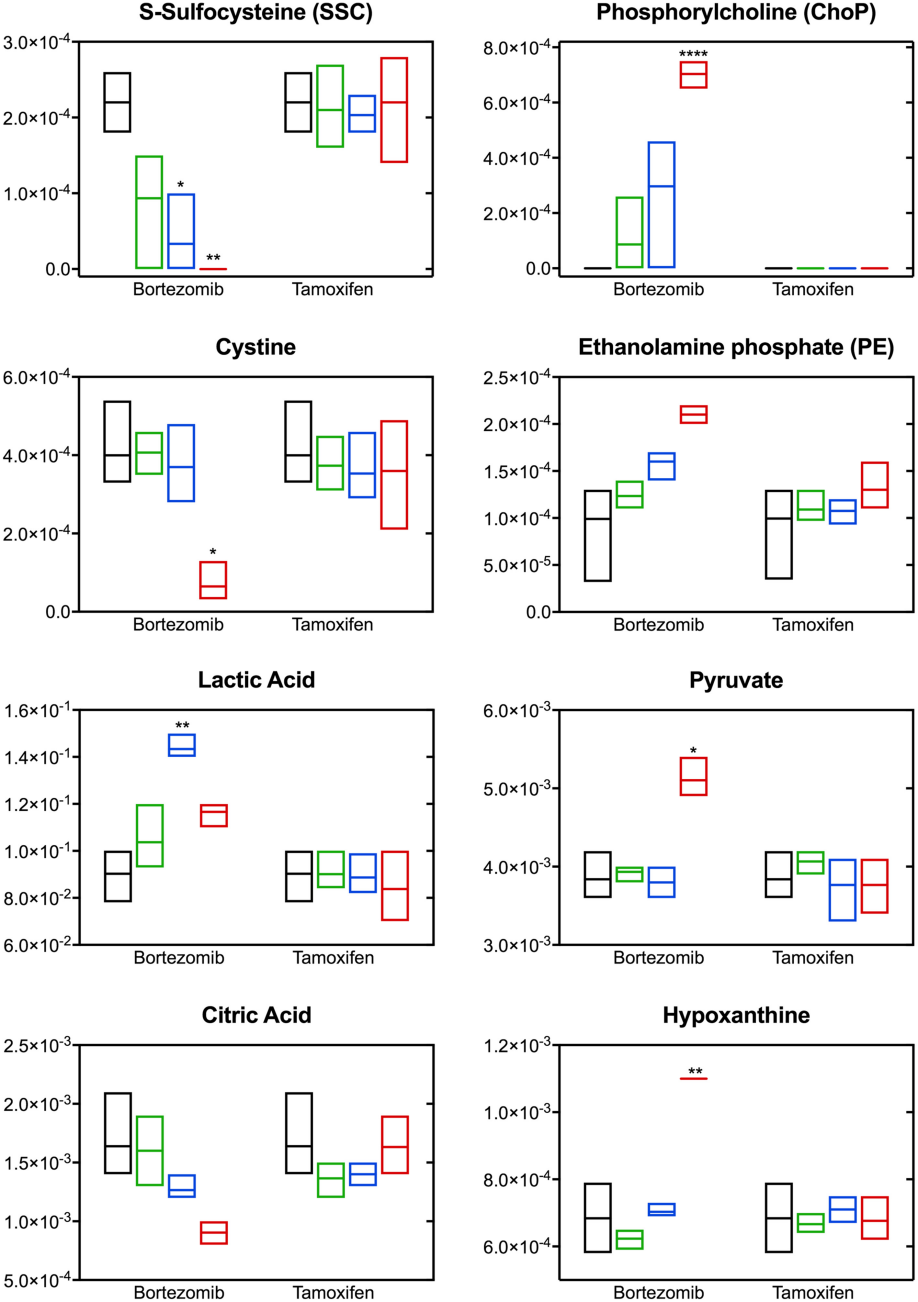
Altered metabolite levels captured 2-days post-treatment with different bortezomib or tamoxifen doses compared to untreated controls. Floating bars (min to max, line at mean) represent relative metabolite abundance for untreated (black, *n* = 5), low-dose treated (green; 0.001 μM bortezomib or 0.01 μM tamoxifen, *n* = 3 for each drug), mid-dose treated (blue; 0.01 μM bortezomib or 0.1 μM tamoxifen, *n* = 3 for each drug), and high-dose treated (red; 0.1 μM bortezomib or 1 μM tamoxifen, *n* = 3 for each drug) brain MPS. Significant alterations (in drug treated samples, with respect to the untreated controls) are marked according to the adjusted *p*-values (multiple *t*-tests with Holm-Sidak correction; ^*^p < 0.05, ^**^p < 0.01, and ^****^p < 0.0001). Non-detected metabolite levels are shown at the baseline (as abundance = 0).

The most significant differences induced with bortezomib treatment were in the relative levels of S-sulfocysteine (SSC) and phosphorylcholine (ChoP) (Figure 5), which are the top-2 metabolites with respect to the calculated VIP scores (metabolites with VIP >1 were considered as the greatest contributors to the group separation) in the PLS-DA model (Supplementary Table 2). Bortezomib had a dose-dependent effect in the levels of SSC and ChoP, whereas these metabolites were unaltered upon tamoxifen treatment. Cystine, the oxidized form of cysteine, was similarly affected by bortezomib treatment, but unaltered by tamoxifen at any dose level. Although these metabolites are on the same canonical metabolic pathway, different dependencies on bortezomib dose were observed. SSC levels gradually decreased with increasing bortezomib concentration, whereas cystine levels were comparable in the untreated, low-dose, and mid-dose treated groups, but significantly lower in the high-dose bortezomib-treated group (Figure 5). Associated with ChoP, ethanolamine phosphate (PE) levels were higher in bortezomib-treated groups, but remained unaltered in tamoxifen-treated groups.

Significant changes were also detected in the levels of lactic acid, pyruvate, citric acid, and hypoxanthine upon bortezomib treatment, but unaltered in the tamoxifen-treated groups (Figure 5). An increase in extracellular levels of lactic acid was observed in the mid-dose bortezomib treated group and the levels in the high-dose bortezomib treated group were similar to that in the untreated controls. Pyruvate levels were comparable in the untreated, low-dose, and mid-dose treated groups, but significantly higher in the high-dose bortezomib treated group. A similar effect on the levels of hypoxanthine, was found, with significantly higher levels in the high-dose bortezomib treated group. Citric acid showed a strong dose-dependent trend to decrease upon bortezomib treatment, and remained unaltered in the tamoxifen-treated groups.

### Bortezomib-Induced Perturbations in Metabolic Pathways Indicate Oxidative Stress and Disruption of Membrane Integrity

**Figure 6:**
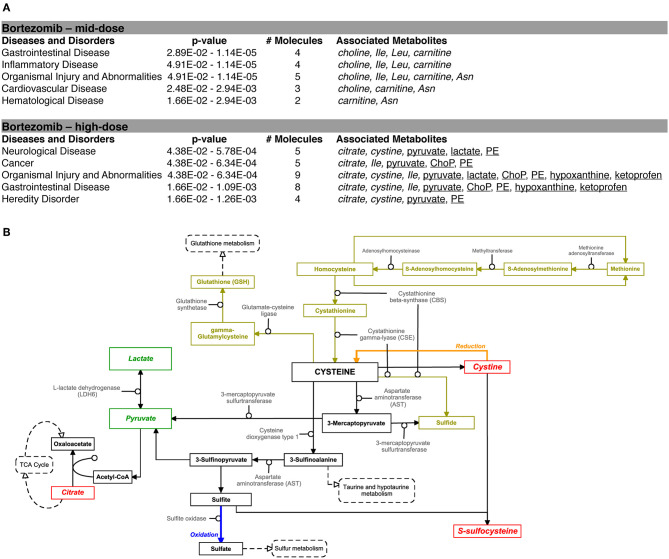
Bortezomib-induced alterations in metabolic pathways. **(A)** Top diseases and disorders list from Ingenuity Pathway Analysis (IPA) for bortezomib mid-dose, and high-dose treatment. Associated decrease or increase in the metabolites are shown in italic or underlined, respectively. **(B)** Bortezomib-induced alterations in the cystine and methionine metabolism. Other metabolic pathways connected to cysteine metabolism are shown in dashed rectangles. Solid rectangles represent metabolites in the pathways and the detected metabolites in this study are shown in italic. Transsulfuration pathway (olive color) shows cysteine synthesis from methionine followed by conversion of cysteine to GSH. Bortezomib-induced decrease or increase in extracellular metabolite levels are highlighted in red and green, respectively.

Pathway analysis using the Ingenuity Pathway Analysis (IPA) software was carried out to examine the importance of altered metabolites in the bortezomib mechanism of action. Only mid-dose and high-dose bortezomib-treated groups were used in comparison to untreated controls, since low-dose bortezomib treatment did not induce significant metabolite alteration. The pathway analysis revealed various diseases and disorders to be associated with the observed metabolite alterations, with “gastrointestinal disease,” and “organismal injury and abnormalities” being common between the mid-dose and high-dose treated groups (Figure 6A). “Neurological disease” and “cancer metabolic pathways,” the only disease and disorder pathways clearly relevant for the brain MPS, were reported to be associated with the metabolite alterations in the high-dose treatment group.

Bortezomib treatment caused a decrease in secreted levels of SSC and cystine (Figure 5), which are both metabolites in cysteine and methionine metabolism (Figure 6B) that regulates redox homeostasis in brain tissue and neurons (McBean, 2017; Paul et al., 2018). Examination of the pathway diagram indicates relationships among measured metabolites as well as informs the mechanism of action of bortezomib in the brain MPS. These results may suggest a correlation in cystine and SSC levels, and possibly disruption of redox balance in the neural constructs upon bortezomib treatment.

Significant elevations in the levels of extracellular phosphorylcholine (ChoP) and ethanolamine phosphate (PE) in bortezomib-treated groups (Figure 5) were also observed. PE and ChoP are in the glycerophospholipids pathway and both showed similar dose-responses to bortezomib treatment. ChoP is a functional group in the hydrophilic head of the phospholipids forming lipid bilayers, and its release is only possible through a leaky membrane (Walter et al., 2004). Detection of ChoP in the culture media of bortezomib-treated group may suggest that this a consequence of bortezomib-induced disruption of membrane integrity.

### Metabolic Signatures Correlate With NAA as Early Indicators of Cellular Dysfunction Followed by DJ-1 Release

Drug-induced response was observed in metabolic signatures and NAA levels 2-days post treatment. While no alterations in DJ-1 levels were detected that quickly, after 4-days of bortezomib treatment, DJ-1 levels were in fact upregulated and NAA levels downregulated.

To relate metabolic signatures from untargeted extracellular metabolomics to the clinically-relevant protein biomarkers, correlation analyses were carried out. For all untreated, bortezomib-treated, and tamoxifen-treated groups, NAA, and DJ-1 levels were analyzed with respect to metabolomics measured on day 16 (2-day drug exposure for drug-treated groups). NAA showed both strong positive and negative correlations with many metabolites (Supplementary Table 3 and Supplementary Figure 2), as expected, since NAA, the neuronal health biomarker is a metabolite in alanine, aspartate, and glutamate metabolism. To verify the NAA-metabolite correlations, a linear regression model was used for each metabolite with correlation coefficient higher than 0.7 (|*r*| > 0.7) and a *p-value* lower than 0.05 (Supplementary Table 3, metabolites in bold); the regression line with 95% confidence interval was mapped over the scatter plot (Supplementary Figure 3).

Similar analyses carried out for DJ-1 (measured on day 16) showed that this oxidative stress biomarker did not have any strong correlations with the metabolic fingerprints on the same time point (Supplementary Table 3 and Supplementary Figure 2). Considering the measured DJ-1 levels on day 18 recapitulated the drug-response more precisely, correlation analyses were also carried out with accumulated DJ-1 levels (total amount measured on days 16 and 18). Interestingly, accumulated DJ-1 was strongly correlated with the metabolites that showed strong correlations with NAA on day 16 (Supplementary Figures 2, 4 and Supplementary Table 3).

**Figure 7:**
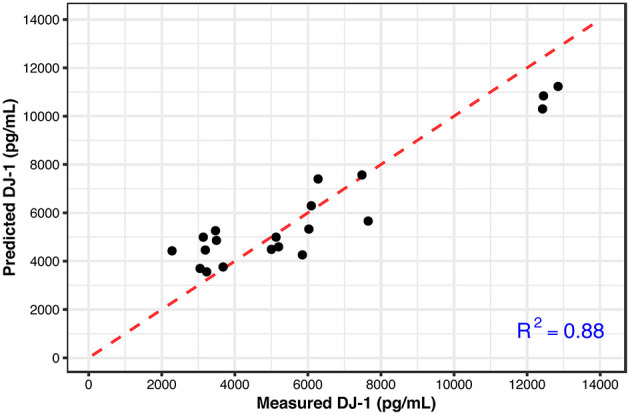
Predicted DJ-1 levels using the Lasso regression model (black circles) with respect to the measured DJ-1 levels. *R*^2^ = 0.88 shows the goodness of fit between the measured and predicted DJ-1 levels. The identity line is shown in red.

These results show that the metabolic signatures and NAA levels correlate well with the measured DJ-1 levels over the longer time period. This finding suggests that a subset of metabolites along with NAA could predict the later DJ-1 levels; the phenotypic outcome indicating cell death induced by cellular dysfunction associated with oxidative stress. To detect this subset, Lasso, a regularized regression method was employed to select the predictors (metabolites) that best define the dependent variable, accumulated DJ-1. The final model constructed at the optimized tuning parameter (λ), selected ethanolamine phosphate (PE), hypoxanthine, and phosphorylcholine (ChoP) as the predictors while filtering out the rest of the metabolites that are not of importance for prediction (Supplementary Table 4 and Supplementary Figure 5). Using this model, accumulated DJ-1 was predicted, and the goodness of fit (*R*^2^) of the prediction was calculated as 0.88 with respect to the measured DJ-1 levels (Figure 7). These results indicate that the measured DJ-1 at the later time point could be predicted using the measured relative abundance values of this subset of metabolites on the earlier time point.

## Discussion

Early assessment of adverse effects of therapeutic drugs in humans is crucial to avoid long-lasting harm. The detection of adverse phenotypic responses of tissues and organs demands continuous monitoring of response biomarkers in humans. Metabolomic biomarkers have the potential to provide early insights about organ- and tissue-level dysfunction prior to irreversible toxicity occurring. In this regard, human MPS technologies, which are designed to be more physiologically-relevant than existing 2D cellular *in vitro* systems, may provide preclinical platforms for drug screening studies. These technologies support long-term tissue culture, enabling the study of kinetics of drug-physiology interactions using various continuous and endpoint metrics, such as clinical biomarkers and -omics. As such, for toxicology, these technologies hold promise not only for identifying toxic effects at the preclinical stage but also providing insights about toxicity mechanisms, identification of biomarkers, and informing clinical biomarker strategies. Similar to the existing 2D *in vitro* models, utilization of the MPS technologies also necessitates assay development such as biomarkers, cell viability/metabolism, and cytotoxicity, in terms of media interference of the assay on detection, assay sensitivity, and molecular stability. Additionally, accurate comparison of *in vitro* systems requires quantitative characterization of each experimental system, e.g., cell population, cell numbers, effect of different medium composition on *in vitro* drug bioavailability, and accuracy of the analytical methods.

This study focuses on drug-induced neurotoxicity using a brain microphysiological system (MPS) treated with known neurotoxic and non-neurotoxic drugs, and assessment using both targeted and untargeted molecular profiling. The untreated brain MPS was characterized using immunocytochemistry imaging and measurement of targeted biomarkers (NAA and DJ-1) over the 28-day cell culture. The immunocytochemistry imaging demonstrated similar mixed neuron-glial cell culture extending around the circumference on days 14 and 28 for untreated controls. Additionally, over the 28-day culture period, NAA and DJ-1 levels gradually increased from the start of the cell culture and reached a plateau after day 14. The drug responses were then evaluated between days 14 and 28 using targeted and untargeted biomarkers.

Enhanced NAA levels were previously found in neuro-pathological conditions, suggesting that measurement of NAA could be means of monitoring neuronal health (Simone et al., 2011; Tortorella et al., 2011). In this study, 2-days of bortezomib treatment induced high levels of NAA release and altered the cells' metabolic fingerprint, suggesting cellular dysfunction. After 4-days of drug treatment, high levels of DJ-1 were also observed, confirming bortezomib-induced oxidative stress. This sequence suggests that neuronal function is impaired quickly, as indicated by NAA, even while overt oxidative stress damaging the cell membrane as indicated by DJ-1 leakage into the medium is only measurable several days later. The untargeted metabolomics results further support these findings, with the elevated levels of PE, a phospholipid breakdown product, and ChoP, a compound released from the cells with leaky membranes. This study suggests that a subset of the detected metabolites in the untargeted metabolomics study following 2-days of treatment could predict the phenotypic outcome (DJ-1) observed at a later time point (after 4-days of treatment). The subset of metabolites that were predictive consisted of ethanolamine phosphate (PE), hypoxanthine, and phosphorylcholine (ChoP). These findings are of particular relevance since NAA and DJ-1, known relevant molecular biomarkers, combined with metabolomics could reveal additional biomarkers to be used in the clinic and could be utilized for assessment of drug-induced neurotoxicity of NMEs.

Bortezomib, used in the treatment of relapsed/refractory multiple myeloma and mantle cell lymphoma, has been reported to induce gastrointestinal toxicity, thrombocytopenia, asthenia, peripheral neuropathy (Menashe, 2007) and have adverse pulmonary effects (Miyakoshi et al., 2006). Exposure to bortezomib causes unfolded proteins to accumulate in the endoplasmic reticulum (ER), causing ER stress and triggering cell stress associated with ROS accumulation (Ri, 2016). Indeed, previous *in vitro* proteasome inhibition studies showed direct mitochondrial function effect, causing accumulation of ubiquitinated proteins within the mitochondrion and leading to increased reactive oxygen production (Sullivan et al., 2004; Torres and Perez, 2008). In the brain, reactive oxygen and nitrogen species are mostly elevated compared to other organs in the body, and the redox balance would normally be regulated via the antioxidants GSH and cysteine. Disruption of this redox homeostasis has been reported to play an important role in progression of neurodegenerative disorders (Shanker and Aschner, 2001; McBean, 2017; Paul et al., 2018).

In this study, bortezomib had a dose-dependent effect on the levels of the metabolites SSC, and cystine, the oxidized form of cysteine, whereas these were unaltered by tamoxifen treatment. SSC, a product of a not well-established cystine-sulfite reaction, was shown to exhibit depolarization (Meweitt et al., 1983), and elevated levels of SSC contributed to neurotoxicity by decreasing intracellular levels of free radicals (Moore et al., 1987). Both SSC and cystine are found in the cysteine metabolism pathway that regulates redox homeostasis in brain tissue and neurons (McBean, 2017; Paul et al., 2018). For the regulation of cysteine metabolism, cells use multiple mechanisms to maintain a constant cysteine supply, either by synthesizing it from methionine via the reverse transsulfuration pathway (Figure 6B), or by the uptake of extracellular cystine via the transporter system ${\text{x}}_{\text{c}}^{-}$. The observed decrease in the extracellular cystine levels in the high-dose bortezomib-treated group may indicate increased cystine uptake from the media (Figure 5 and Supplementary Figure 1). The ${\text{x}}_{\text{c}}^{-}$ system transports extracellular cystine in exchange with glutamate. In this study, extracellular glutamate levels were detected only for the high-dose bortezomib treated group (Supplementary Figure 1), in accordance with the lower extracellular cystine levels. Similar findings were reported suggesting that depletion of extracellular cyst(e)ine triggers oxidative stress via depletion of intracellular GSH, followed by cell cycle arrest and cell death (Cramer et al., 2017).

Oxidative stress or disruption of redox homeostasis in general has been associated with neurodegenerative diseases, due to change in the glutamate levels in the brain, causing calcium influx into neurons, eventually leading to breakdown of membrane structural elements (Klein, 2000). In this context, phospholipids, the essential structural elements of the cell membrane, were reported to be elevated in the cerebrospinal fluid of Alzheimer patients (Walter et al., 2004). Our results in the brain MPS were similar, where bortezomib treatment induced significant elevations in both extracellular ChoP and PE. PE, a precursor of phospholipid synthesis, is a phospholipid breakdown product, and contributes to the sphingolipid metabolism. ChoP is a functional group in the hydrophilic head of the phospholipids forming the lipid bilayers and release of this compound from the cell is possible through a leaky membrane (Walter et al., 2004). These observations may suggest release of ChoP to the culture media is a consequence of bortezomib-induced disruption of membrane integrity. For untreated and tamoxifen treated groups, ChoP was undetectable in the MPS medium. ChoP normally can be hydrolyzed to free choline in the cell and be released through certain transporters (Klein, 2000). However, changes in the extracellular choline levels are not specific to disruption of membrane integrity since choline could be hydrolyzed from (or condensed to) acetylcholine, or oxidized to betaine which provides methyl groups to form methionine in the transsulfuration pathway of the cysteine and methionine metabolism. In this study, a decrease in the extracellular choline levels for the low-dose and mid-dose bortezomib treated groups was observed, but the levels were comparable for the high-dose bortezomib, tamoxifen treated, and untreated groups (Supplementary Figure 1). Hence, an increased ChoP release could be an indicator of membrane breakdown, but not the only driving force of the change in the extracellular choline levels.

Significant changes in the levels of lactic acid, pyruvate, citric acid, and hypoxanthine were detected upon bortezomib treatment but not with tamoxifen. The functions of several of these may give clues to the mechanisms by which bortezomib is adversely affecting the tissue. Hypoxanthine is a purine derivative commonly utilized as a hypoxia biomarker (Saugstad, 1988), while citric acid is a key constituent in the TCA cycle. While the changes observed in the citrate levels may be correlated with pyruvate (and lactate) through the TCA cycle, previous studies reported that citrate is synthesized in and released from astrocytes in large amounts, which contributes to its regulatory function in the CNS as an extracellular chelating agent (Westergaard et al., 2017). Moreover, bortezomib-induced perturbations in these metabolites may also suggest mitochondrial dysfunction of the neural constructs.

For the statistical analysis of the metabolomics data, multiple *t*-tests were carried out and Holm-Sidak correction was applied to account for multiple comparisons problem. The Sidak method allows strong control on the familywise error rate (FWER), and it is believed to be too conservative in some cases. False discovery rate (FDR) measure may be more reasonable since it allows a proportion of Type I errors to occur. When the statistical analyses were repeated with FDR measure (with Q = 5%), our results were the same for tamoxifen treatments, and low- and mid-dose bortezomib treatments, while the number of significantly altered metabolites upon high-dose bortezomib treatment were higher (data not shown). Metabolites such as citric acid, and PE, which were not significant in the Holm-Sidak method were significant in the FDR method. Considering that Sidak correction might be too conservative, the metabolites with lower adjusted-*p*-values (0.05–0.1) were already included in our biological interpretation of the metabolomics data.

In summary, clinically relevant biomarkers for neural health and neurotoxic response were quantified in the brain MPS. The changes in biomarker profiles upon neurotoxic drug treatment using both targeted and untargeted molecular biomarkers indicated oxidative stress and membrane breakdown in the brain MPS. Furthermore, a subset of early response biomarkers identified with untargeted metabolomics after a first dose of neurotoxic drug administration was correlated to DJ-1 protein levels, which was observed after repeated drug dosing. These results indicated that early response biomarkers could be used to predict adverse drug effects in repeated drug dosing regimens before overt cell death occurs. These clinically relevant biomarkers could also be used to assess early response in humans to avoid irreversible drug-induced toxicity. While our study does not provide a deep biological characterization of the brain MPS and its relevance to the human brain in terms of tissue/cell content and organ function, we believe this study to be a proof of concept for the assessment of preclinical response biomarkers and utilization of a 3D *in vitro* model for possible drug screening studies. Under the conditions used in the study, we found that NAA, DJ-1, and extracellular untargeted metabolomics were able to capture a differential response for a neurotoxic, and a non-neurotoxic drug. It should be noted that response biomarkers are highly dependent on the drug mechanism of action (MoA), and should be chosen with care for large scale screening of drugs that belong to different classes of MoA. We acknowledge the limitations of the brain MPS, sensitivity of the NAA, DJ-1, and untargeted metabolomics measurements, and the drugs used in this study as they provide an individual case study rather than demonstrating a high throughput drug screening or prediction of neurotoxicity. However, we suggest that the approach demonstrated here can be extended for preclinical toxicity screening and biomarker discovery studies.

## Author Contributions

WM and JT developed the brain microphysiological system and provided tissue culture protocols. SM and MC planned the experiments. SM performed the experiments, analyzed the data, and wrote the main manuscript text. BA performed the metabolomics analysis and wrote the main manuscript text. CS and MC directed the work and edited the manuscript. All authors reviewed the manuscript.

### Conflict of Interest Statement

WM is a co-founder and stockholder in Stem Pharm, Inc. MC is a co-founder and stockholder in Javelin Biotech, Inc. CS is employed by the company Stokes Consulting. The remaining authors declare that the research was conducted in the absence of any commercial or financial relationships that could be construed as a potential conflict of interest.
