# Supplementary material for: Assessment of Drug-Induced Toxicity Biomarkers in the Brain Microphysiological System (MPS) Using Targeted and Untargeted Molecular Profiling
Source: Front Big Data. 2019 Jun 26;2:23. doi: 10.3389/fdata.2019.00023 (PMC7931859; doi:10.3389/fdata.2019.00023)
Supplement: Supplementary file 1 [file Data_Sheet_1.zip › Supplementary_Materials/Supplementary_Materials.docx]

Supplementary Material

**Assessment of drug-induced toxicity biomarkers in the brain microphysiological system (MPS) using targeted and untargeted molecular profiling**

**Sara G. Mina^1†^, Begum Alaybeyoglu^1†^, William L. Murphy^2^, James A. Thomson^3,4^, Cynthia L. Stokes^5^, Murat Cirit^1*^**

^1^ Department of Biological Engineering, Massachusetts Institute of Technology, Cambridge, MA, United States

^2^ Department of Biomedical Engineering, University of Wisconsin-Madison, WI, United States

^3^ Regenerative Biology, Morgridge Institute for Research, Madison, WI, United States

^4^ Department of Cell and Regenerative Biology, University of Wisconsin Madison, WI, United States

^5^ Stokes Consulting, Redwood City, CA, United States

**†** Authors contributed equally

*** Correspondence:** Murat Cirit murat@javelinbio.com


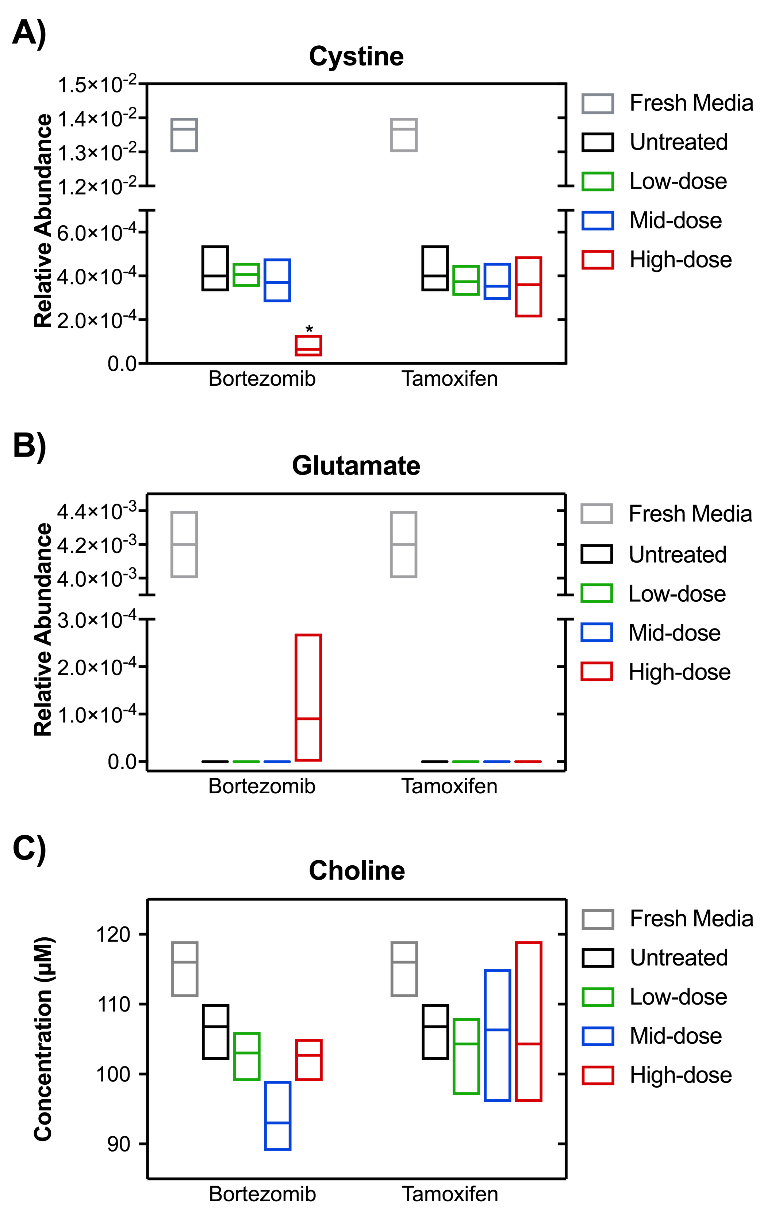


**Supplementary Figure 1.** Altered metabolite levels captured 2-days post-treatment with different bortezomib or tamoxifen doses compared to untreated controls. Floating bars (min to max, line at mean) represent relative metabolite abundance for fresh media (gray, n = 3), untreated (black, n = 5), low-dose treated (green; 0.001 µM bortezomib or 0.01 µM tamoxifen, n = 3 for each drug), mid-dose treated (blue; 0.01 µM bortezomib or 0.1 µM tamoxifen, n = 3 for each drug), and high-dose treated (red; 0.1 µM bortezomib or 1 µM tamoxifen, n = 3 for each drug) neural constructs. Significant alterations (in drug treated samples, with respect to the untreated controls) are marked according to the adjusted p-values (multiple t tests with Holm-Sidak correction; *p < 0.05). Non-detected metabolite levels are shown at the baseline (as abundance = 0). **(A)** Extracellular cystine is almost completely consumed in the high-dose bortezomib-treated group. **(B)** Glutamate was detected only in the fresh media, and high-dose bortezomib-treated groups. **(C)** Choline levels were relatively low in the mid-dose bortezomib-treated group compared to the untreated and tamoxifen-treated groups.


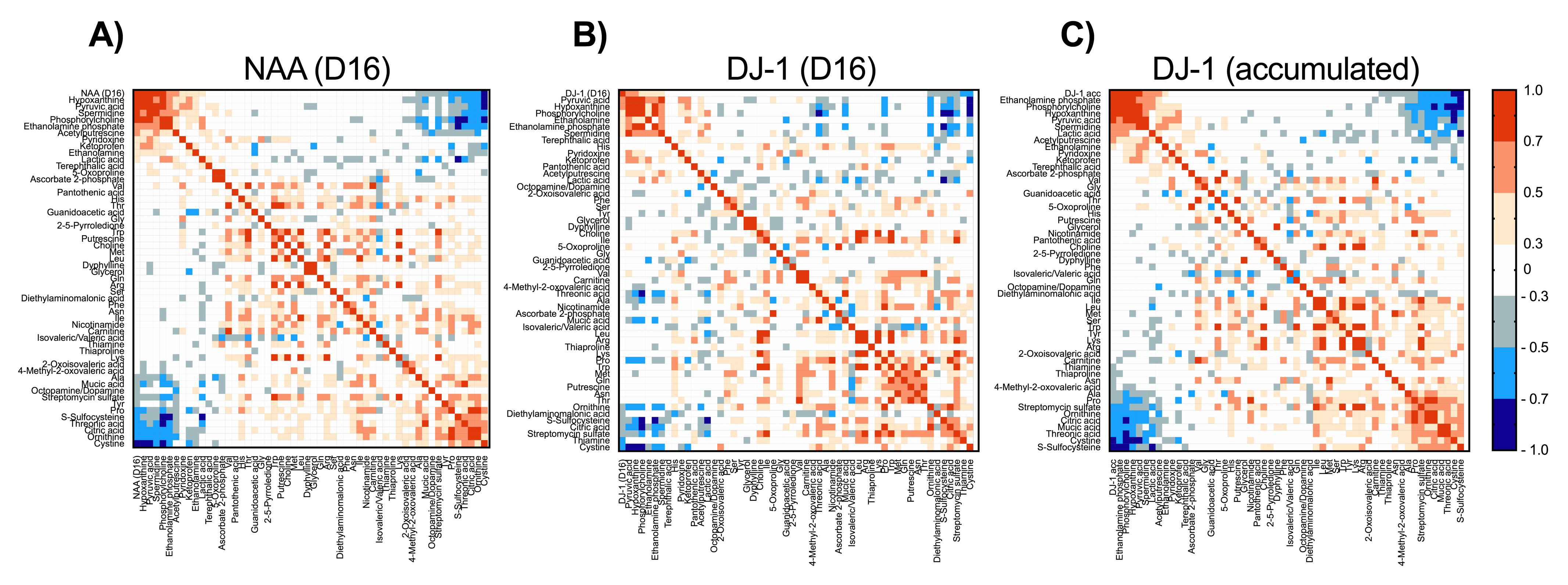


**Supplementary Figure 2.** Correlation matrices for **(A)** NAA on day 16, **(B)** DJ-1 on day 16, and **(C)** accumulated DJ-1 (total amounts on day 16 and day 18) with untargeted metabolomics. All untreated, bortezomib-treated, and tamoxifen-treated samples were used for analysis that consisted of n=23 for NAA and n=20 for DJ-1 datasets. Positive correlations are shown in orange (from dark to light orange, strong to weak correlations with Pearson’s r > 0.7, r > 0.5, and r > 0.3, respectively), negative correlations are shown in blue (from dark to light blue, strong to weak correlations with Pearson’s r < -0.7, r < -0.5, and r < -0.3, respectively), and white shows the biomarker-metabolite couples with no linear correlation.


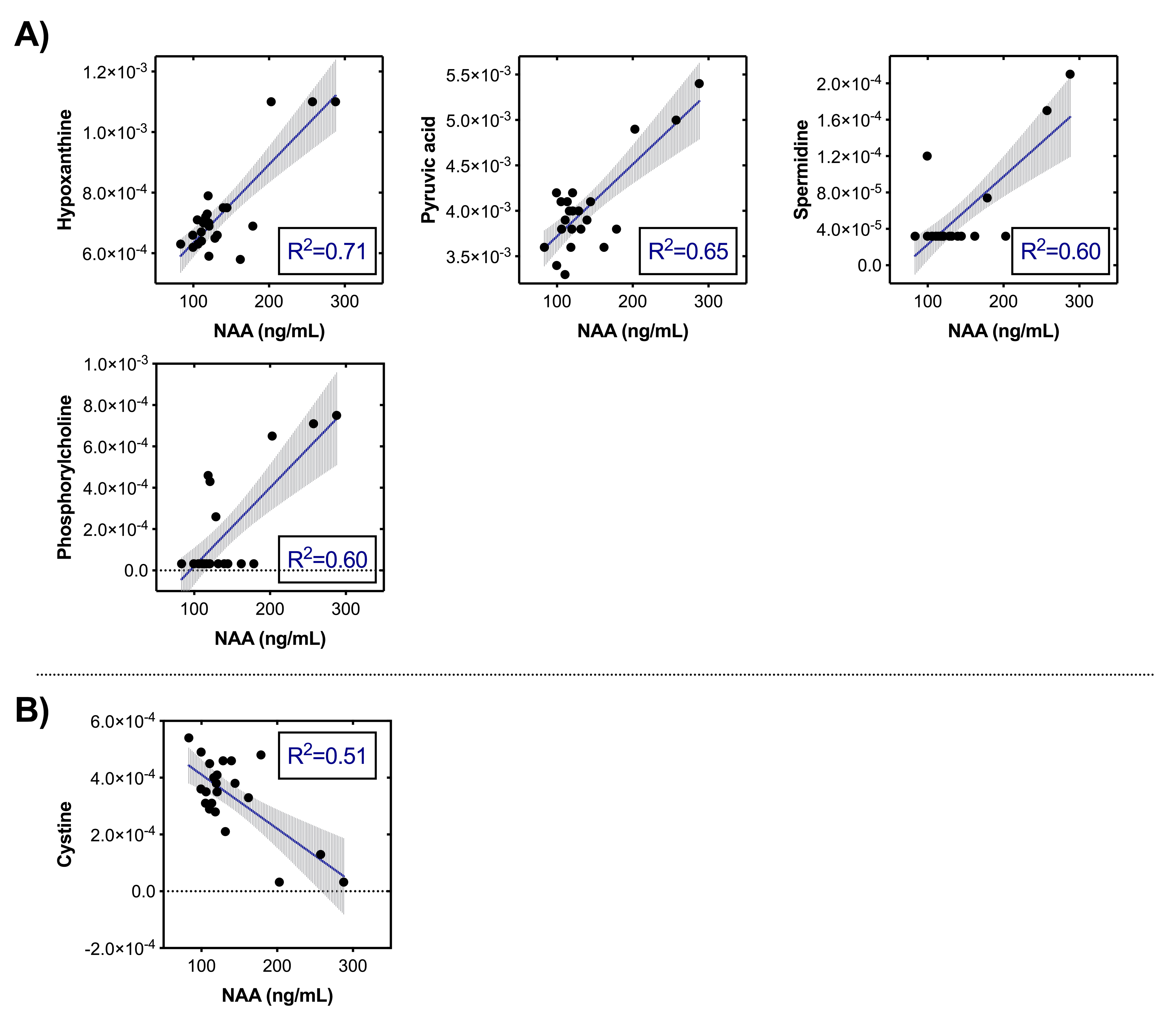


**Supplementary Figure 3.** Simple linear regression (SLR) plots for the correlated metabolites with NAA (day 16). **(A)** Hypoxanthine, pyruvate, spermidine, and phosphorylcholine (ChoP) showed strong positive correlations with NAA. **(B)** Cystine showed strong negative correlation with NAA. Regression lines (blue), and 95% confidence interval regions (gray error bars) are shown as overlapped with data (scatter, black) to demonstrate the fit for each SLR. The coefficient of determination (R^2^) for each model is shown in the insets. Metabolite levels are measured and reported as relative abundance, and NAA levels are measured in ng/mL.


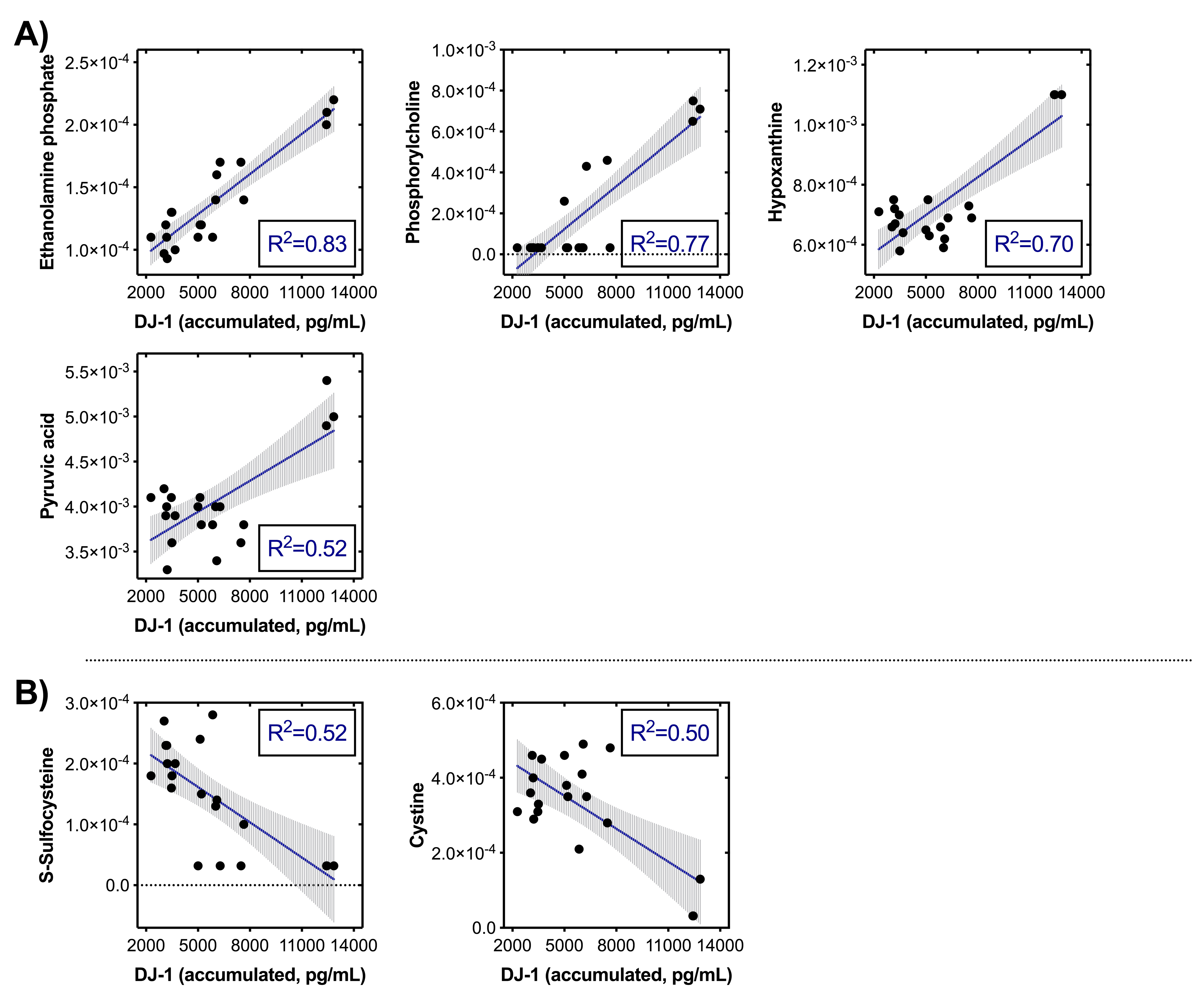


**Supplementary Figure 4.** Simple linear regression (SLR) plots for the correlated metabolites with accumulated DJ-1 (total of the measured amounts on day 16 and day 18). **(A)** Ethanolamine phosphate (PE), phosphorylcholine (ChoP), hypoxanthine, and pyruvate showed strong positive correlations with DJ-1. **(B)** S-sulfocysteine (SSC), and cystine showed strong negative correlations with DJ-1. Regression lines (blue), and 95% confidence interval regions (gray error bars) are shown as overlapped with data (scatter, black) to demonstrate the fit for each SLR. The coefficient of determination (R^2^) for each model is shown in the insets. Metabolite levels are measured and reported as relative abundance, and DJ-1 levels are measured in pg/mL.


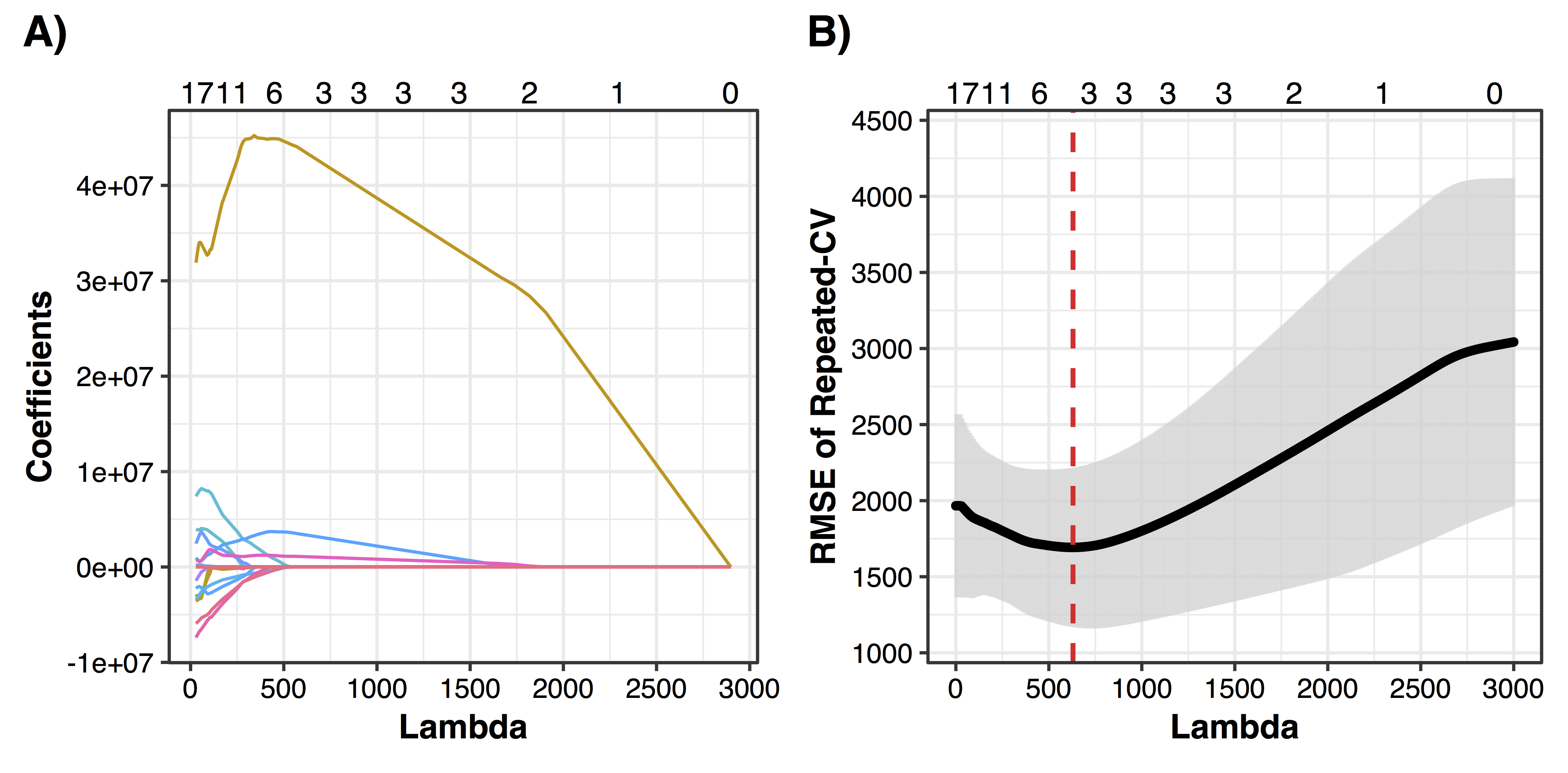


**Supplementary Figure 5.** Lasso regression model was employed for prediction of accumulated DJ-1 (dependent variable) from metabolomics data (intercorrelated predictors) measured on day 16. All untreated (2 samples), bortezomib treated (3 samples for each dose, total of 9), and tamoxifen treated (3 samples for each dose, total of 9) samples were used for model construction (n=20). Metabolites that were not detected (N.D.) in the majority of the samples were removed from the dataset, and a total of 54 metabolites were used in the model as (correlated) predictors. **(A)** Lasso shrinkage method assigns zero to the coefficients of the predictors (X_i_) that are not important for the model, and feature selection was carried out by tuning the parameter lambda (λ). Calculated regression coefficients (β_i_) are shown at each λ, where each line corresponds to a metabolite (predictor). **(B)** Root mean square error (RMSE) was calculated using 5-fold cross-validation (CV) with 100 repetitions (standard deviation in gray), and the minimum RMSE was used to detect the optimum lambda value (red vertical line) and the associated coefficients (β_i_) to construct the model. The axis above indicates the number of non-zero coefficients (effective degrees of freedom; *df*) at the corresponding λ.

## Supplementary Tables

Supplementary Table 1. Targeted biomarker measurements upon bortezomib and tamoxifen treatment for 14 days of the brain MPS. DJ-1/PARK7 and N-acetyl-aspartate (NAA) concentrations were measured in the media from each well every 2 days. Day 14 indicates the start of drug treatment. Data shown are mean ± SD (n=3) values for each biomarker. Significant alterations are marked according to the calculated p-values (two-way ANOVA multiple comparisons with a Bonferroni’s post-hoc test; *p < 0.05, **p < 0.01, ***p < 0.001, and ****p < 0.0001). BLOQ represents *Below Limit of Quantification* (5 ng/mL) for NAA measurement.

| **Drug** | **Dose (µM)** | **Day** | **DJ-1 (mg/mL)** Mean±SD | **Significant** | **Adjusted p Value** | **NAA (ng/mL)** Mean±SD | **Significant** | **Adjusted p Value** |
| --- | --- | --- | --- | --- | --- | --- | --- | --- |
|  |  |  |  |  |  |  |  |  |
| **Bortezomib** | 0 | 14 | 7.8E02 ± 7.9E01 |  |  | 9.6E01 ± 1.9E01 |  |  |
|  |  | 16 | 1.1E03 ± 2.9E02 |  |  | 1.4E02 ±2.4E01 |  |  |
|  |  | 18 | 2.2E03 ± 1.1E02 |  |  | 2.1E02 ± 3.2E01 |  |  |
|  |  | 20 | 1.4E03 ± 9.9E02 |  |  | 1.5E02 ± 2.5E01 |  |  |
|  |  | 22 | 1.7E03 ± 2.1E02 |  |  | 1.9E02 ± 2.7E00 |  |  |
|  |  | 24 | 1.4E03 ± 3.1E02 |  |  | 1.6E02 ± 1.5E01 |  |  |
|  |  | 26 | 1.8E03 ± 1.7E02 |  |  | 1.7E02 ± 1.1E01 |  |  |
|  |  | 28 | 1.2E03 ± 2.2E02 |  |  | 1.6E02 ± 1.6E01 |  |  |
|  | 0.001 | 14 | 1.4E03 ± 1.9E02 | No | >0.9999 | 9.7E01 ± 1.7E01 | No | 0.9743 |
|  |  | 16 | 1.4E03 ± 1.5E02 | No | >0.9999 | 1.2E02 ± 1.1E01 | No | <0.0001 |
|  |  | 18 | 4.0E03 ± 4.1E02 | No | 0.0555 | 1.9E02 ± 3.7E01 | No | <0.0001 |
|  |  | 20 | 1.6E03 ± 2.9E02 | No | >0.9999 | 1.2E02 ± 4.5E00 | No | <0.0001 |
|  |  | 22 | 3.0E03 ± 1.5E03 | No | 0.3121 | 1.9E02 ± 1.9E02 | No | <0.0001 |
|  |  | 24 | 1.3E03 ± 1.1E03 | No | >0.9999 | 1.2E02 ± 8.8E01 | No | <0.0001 |
|  |  | 26 | 2.4E03 ± 3.6E02 | No | >0.9999 | 1.1E02 ± 9.1E01 | No | <0.0001 |
|  |  | 28 | 3.3E03 ± 2.2E03 | Yes | 0.0208 | 1.3E02 ± 1.1E02 | No | <0.0001 |
|  | 0.01 | 14 | 1.4E03 ± 1.9E02 | No | >0.9999 | 1.1E02 ± 2.2E00 | No | >0.9999 |
|  |  | 16 | 9.9E02 ± 9.3E01 | No | >0.9999 | 1.E02 ± 3.4E01 | No | >0.9999 |
|  |  | 18 | 6.1E03 ± 7.1E02 | Yes | <0.0001 | 2.8E01 ± 6.7E00 | Yes | <0.0001 |
|  |  | 20 | 6.4E03 ± 1.1E03 | Yes | <0.0001 | 1.2E02 ± 1.5E01 | No | 0.6675 |
|  |  | 22 | 5.7E03 ± 7.2E02 | Yes | <0.0001 | 1.3E02 ± 2.3E01 | Yes | 0.0045 |
|  |  | 24 | 2.7E03 ± 6.3E02 | Yes | 0.0171 | 1.2E02 ± 2.0E01 | No | 0.1766 |
|  |  | 26 | 3.5E03 ± 3.9E02 | Yes | 0.0012 | 1.0E02 ± 1.5E01 | Yes | 0.0032 |
|  |  | 28 | 2.4E03 ± 1.3E02 | Yes | 0.0469 | 1.1E02 ± 1.7E01 | Yes | 0.0155 |
|  | 0.1 | 14 | 1.1E03 ± 4.1E01 | No | >0.9999 | 1.2E02 ± 2.9E01 | No | 0.9743 |
|  |  | 16 | 1.8E03 ± 3.1E02 | No | 0.1223 | 2.5E02 ± 4.3E01 | Yes | <0.0001 |
|  |  | 18 | 1.1E04 ± 5.2E02 | Yes | <0.0001 | 7.9E01 ± 4.9E00 | Yes | <0.0001 |
|  |  | 20 | 3.2E03 ± 5.1E02 | Yes | <0.0001 | BLOQ | Yes | <0.0001 |
|  |  | 22 | 1.9E03 ± 3.9E02 | No | >0.9999 | BLOQ | Yes | <0.0001 |
|  |  | 24 | 3.7E02 ± 7.7E01 | Yes | 0.0148 | BLOQ | Yes | <0.0001 |
|  |  | 26 | 2.3E02 ± 4.0E01 | Yes | 0.0001 | BLOQ | Yes | <0.0001 |
|  |  | 28 | 5.0E01 ± 6.5E01 | Yes | 0.0034 | BLOQ | Yes | <0.0001 |
| **Tamoxifen** | 0.01 | 14 | 1.3E03 ± 7.0E01 | No | >0.9999 | 8.8E01 ± 1.3E01 | No | >0.9999 |
|  |  | 16 | 1.2E03 ± 8.8E01 | No | >0.9999 | 1.1E02 ± 7.5E00 | No | >0.9999 |
|  |  | 18 | 2.2E03 ± 2.4E02 | No | 0.0555 | 1.4E02 ± 4.3E01 | No | 0.0544 |
|  |  | 20 | 1.3E03 ± 5.3E02 | No | >0.9999 | 1.2E02 ± 3.1E01 | No | >0.9999 |
|  |  | 22 | 2.2E02 ± 1.0E03 | No | 0.3121 | 1.7E02 ± 2.5E01 | No | >0.9999 |
|  |  | 24 | 1.6E03 ± 1.7E02 | No | >0.9999 | 1.1E02 ± 5.2E01 | No | 0.5781 |
|  |  | 26 | 1.7E03 ± 5.4E02 | No | >0.9999 | 1.0E02 ± 3.9E01 | No | 0.1179 |
|  |  | 28 | 1.6E03 ± 1.6E02 | Yes | 0.0208 | 9.6E01 ± 6.7E01 | No | 0.1535 |
|  | 0.1 | 14 | 1.4E03 ± 1.2E02 | No | >0.9999 | 8.9E01 ± 2.3E01 | No | >0.9999 |
|  |  | 16 | 5.8E02 ± 1.6E02 | No | >0.9999 | 1.2E02 ± 1.8E01 | No | >0.9999 |
|  |  | 18 | 2.3E03 ± 4.2E02 | Yes | <0.0001 | 1.7E02 ± 2.1E01 | No | 0.297 |
|  |  | 20 | 7.0E02 ± 1.3E02 | Yes | <0.0001 | 1.1E02 ± 1.5E01 | No | 0.078 |
|  |  | 22 | 1.6E03± 1.5E02 | Yes | <0.0001 | 1.5E02 ± 3.6E01 | No | 0.2235 |
|  |  | 24 | 1.1E03 ± 9.0E01 | Yes | 0.0171 | 1.7E02 ± 8.9E00 | No | >0.9999 |
|  |  | 26 | 1.9E03 ± 3.2E02 | Yes | 0.0012 | 1.7E02 ± 1.1E01 | No | >0.9999 |
|  |  | 28 | 6.8E02 ± 2.3E02 | Yes | 0.0469 | 1.6E02 ± 2.8E01 | No | >0.9999 |
|  | 1 | 14 | 1.7E03 ± 6.4E02 | No | >0.9999 | 9.5E01 ± 2.2E01 | No | >0.9999 |
|  |  | 16 | 1.3E03 ± 4.5E02 | No | 0.1223 | 1.3E02 ± 2.3E01 | No | >0.9999 |
|  |  | 18 | 4.4E03 ± 9.4E02 | Yes | <0.0001 | 2.2E02 ± 5.8E01 | No | >0.9999 |
|  |  | 20 | 1.1E03 ± 2.9E02 | Yes | <0.0001 | 1.0E02 ± 1.4E01 | No | 0.1018 |
|  |  | 22 | 2.9E03 ± 4.5E02 | No | >0.9999 | 1.5E02 ± 2.2E01 | No | 0.2089 |
|  |  | 24 | 1.7E03 ± 3.2E02 | Yes | 0.0148 | 1.4E02 ± 7.0E01 | No | >0.9999 |
|  |  | 26 | 1.7E03 ± 4.6E02 | Yes | 0.0001 | 1.1E02 ± 7.1E00 | Yes | 0.0425 |
|  |  | 28 | 8.4E02 ± 7.4E01 | Yes | 0.0034 | 1.0E02 ± 1.7E01 | Yes | 0.0362 |

Supplementary Table 2. PLS-DA models employed for separation of bortezomib or tamoxifen treatment from untreated controls. (top) PLS-DA diagnostics for the bortezomib and tamoxifen models. Bortezomib model has high R2 and Q2 values relatively comparable for 2 components and 3 components. Tamoxifen model has negative Q2 for up to 8 components suggesting that the model is not predictive (or is overfitted). (bottom) List of important features in the bortezomib model identified by their Variable Importance in Projection (VIP) scores for the first 3 components. Metabolites with a VIP score > 1.0 are considered important features in the projection.

| **PLS-DA Diagnostics** | | | | | | | | | |
| --- | --- | --- | --- | --- | --- | --- | --- | --- | --- |
|  | **Bortezomib** | | | **Tamoxifen** | | | | | |
|  | **1 comp** | **2 comps** | **3 comps** | **1 comp** | | **2 comps** | **3 comps** | | **8 comps** |
| **Q^2^** | 0.71 | 0.79 | 0.80 | -0.74 | | -0.76 | -0.51 | | -0.56 |
| **R^2^** | 0.88 | 0.97 | 0.99 | 0.67 | | 0.93 | 0.98 | | 1.0 |
| **Accuracy** | 0.36 | 0.64 | 0.86 | 0.14 | | 0.14 | 0.07 | | 0.14 |
|  |  |  |  |  | |  |  | |  |
| **VIP Scores in the Bortezomib Model** | | | | | | | | | |
| **Metabolite** | | | **Comp 1** | | **Comp 2** | | | **Comp 3** | |
| Phosphorylcholine (ChoP) | | | 1.91 | | 1.83 | | | 1.81 | |
| S-Sulfocysteine (SSC) | | | 1.91 | | 1.87 | | | 1.86 | |
| Ethanolamine phosphate (PE) | | | 1.87 | | 1.80 | | | 1.78 | |
| Threonic acid | | | 1.80 | | 1.73 | | | 1.71 | |
| Citric acid | | | 1.69 | | 1.62 | | | 1.61 | |
| Streptomycin sulfate | | | 1.67 | | 1.60 | | | 1.59 | |
| Mucic acid | | | 1.65 | | 1.59 | | | 1.57 | |
| Hypoxanthine | | | 1.63 | | 1.55 | | | 1.54 | |
| Cystine | | | 1.59 | | 1.52 | | | 1.50 | |
| Ile | | | 1.55 | | 1.48 | | | 1.47 | |
| Pyruvic acid | | | 1.54 | | 1.48 | | | 1.47 | |
| Lactic acid | | | 1.48 | | 1.46 | | | 1.45 | |
| Ornithine | | | 1.39 | | 1.35 | | | 1.34 | |
| Spermidine | | | 1.37 | | 1.31 | | | 1.31 | |
| Ketoprofen | | | 1.28 | | 1.23 | | | 1.22 | |
| Pro | | | 1.23 | | 1.24 | | | 1.23 | |
| Lys | | | 1.09 | | 1.04 | | | 1.03 | |
| Ala | | | 1.06 | | 1.12 | | | 1.10 | |
| Asn | | | 1.06 | | 1.04 | | | 1.10 | |

Supplementary Table 3. Results of the correlation analyses for NAA (day 16), DJ-1 (day 16), accumulated DJ-1 (total amounts on day 16 and day 18) with untargeted metabolomics. All untreated, bortezomib treated, and tamoxifen treated samples were used for analysis that consisted of n=23 for NAA and n=20 for DJ-1 datasets. Strong, and moderate positive, and negative correlated metabolites are listed with their corresponding Pearson correlation coefficient (r) and significance (p-value) values. Highly correlated metabolites (with |r| > 0.7 and p < 0.05) are shown in bold.

| **NAA (D16)** | | | **DJ-1 (D16)** | | | | **DJ-1 (accumulated, D16+D18)** | | | |
| --- | --- | --- | --- | --- | --- | --- | --- | --- | --- | --- |
| Metabolite | Pearson’s r | p-value | Metabolite | Pearson’s r | p-value | Metabolite | | Pearson’s r | p-value |  |
| **Hypoxanthine** | **0.843** | **4.45E-07** | Pyruvic acid | 0.617 | 3.73E-03 | **Ethanolamine phosphate** | | **0.914** | **1.86E-08** |  |
| **Pyruvic acid** | **0.804** | **3.78E-06** | Hypoxanthine | 0.515 | 2.02E-02 | **Phosphorylcholine** | | **0.877** | **3.98E-07** |  |
| **Spermidine** | **0.773** | **1.50E-05** |  |  |  | **Hypoxanthine** | | **0.836** | **4.35E-06** |  |
| **Phosphorylcholine** | **0.772** | **1.59E-05** |  |  |  | **Pyruvic acid** | | **0.721** | **3.36E-04** |  |
| Ethanolamine phosphate | 0.680 | 3.60E-04 |  |  |  | Spermidine | | 0.612 | 4.11E-03 |  |
| Acetylputrescine | 0.539 | 7.93E-03 |  |  |  | Lactic acid | | 0.531 | 1.59E-02 |  |
|  |  |  |  |  |  | Acetylputrescine | | 0.501 | 2.44E-02 |  |
| Pro | -0.504 | 1.43E-02 | Thiamine | -0.502 | 2.40E-02 | Streptomycin sulfate | | -0.570 | 8.63E-03 |  |
| S-Sulfocysteine | -0.527 | 9.69E-03 | Cystine | -0.531 | 1.59E-02 | Ornithine | | -0.596 | 5.55E-03 |  |
| Threonic acid | -0.583 | 3.51E-03 |  |  |  | Citric acid | | -0.602 | 4.97E-03 |  |
| Citric acid | -0.604 | 2.26E-03 |  |  |  | Mucic acid | | -0.637 | 2.54E-03 |  |
| Ornithine | -0.698 | 2.14E-04 |  |  |  | Threonic acid | | -0.692 | 7.20E-04 |  |
| **Cystine** | **-0.715** | **1.27E-04** |  |  |  | **Cystine** | | **-0.703** | **5.41E-04** |  |
|  |  |  |  |  |  | **S-Sulfocysteine** | | **-0.719** | **3.51E-04** |  |

Supplementary Table 4. Non-zero regression coefficients (β_i_) for predictors (X_i_) at the corresponding minimum root mean-squared error (RMSE_min_) of the 5-fold cross-validation with 100 repetitions. Model with the λ of 630 (at the RMSE_min_ of 1693) has 0.83 of fraction deviance explained (dev). PE has the largest positive coefficient suggesting its importance in prediction. Hypoxanthine and ChoP also have positive coefficients showing their contribution to the prediction.

| **Lasso Model for DJ-1 Prediction** | | |
| --- | --- | --- |
| **Lambda (λ)** |  | 630 |
| **RMSE_min_** |  | 1693 |
| **dev** |  | 0.83 |
| **Coefficient (β_i_)** | | |
| **Intercept** |  | -2.72E+03 |
| **X_1_** | Ethanolamine phosphate (PE) | 4.33E+07 |
| **X_2_** | Hypoxanthine | 3.33E+06 |
| **X_3_** | Phosphorylcholine (ChoP) | 1.07E+06 |
